# Phenotypic characterisation of regulatory T cells in dogs reveals signature transcripts conserved in humans and mice

**DOI:** 10.1038/s41598-019-50065-8

**Published:** 2019-09-17

**Authors:** Ying Wu, Yu-Mei Chang, Anneliese J. Stell, Simon L. Priestnall, Eshita Sharma, Michelle R. Goulart, John Gribben, Dong Xia, Oliver A. Garden

**Affiliations:** 1 Royal Veterinary College, London, UK; 2 Wellcome Centre for Human Genetics, University of Oxford, Oxford, UK; 3 Barts Cancer Institute, Queen Mary University of London, London, UK; 4 School of Veterinary Medicine, University of Pennsylvania, Philadelphia, PA USA; 5 Present Address: School of Veterinary Medicine, University of Pennsylvania, Philadelphia, PA USA; 6 Present Address: Barts Cancer Institute, Queen Mary University of London, London, UK

**Keywords:** Lymphocytes, Immunology

## Abstract

Regulatory T cells (Tregs) are a double-edged regulator of the immune system. Aberrations of Tregs correlate with pathogenesis of inflammatory, autoimmune and neoplastic disorders. Phenotypically and functionally distinct subsets of Tregs have been identified in humans and mice on the basis of their extensive portfolios of monoclonal antibodies (mAb) against Treg surface antigens. As an important veterinary species, dogs are increasingly recognised as an excellent model for many human diseases. However, insightful study of canine Tregs has been restrained by the limited availability of mAb. We therefore set out to characterise CD4^+^CD25^high^ T cells isolated *ex vivo* from healthy dogs and showed that they possess a regulatory phenotype, function, and transcriptomic signature that resembles those of human and murine Tregs. By launching a cross-species comparison, we unveiled a conserved transcriptomic signature of Tregs and identified that transcript *hip1* may have implications in Treg function.

## Introduction

Regulatory T cells (Tregs) are dominant regulators of immune responses against self, pathogenic and commensal antigens in the periphery^1^. As key players in the maintenance of immune health, aberrations of Tregs have pathogenic implications in a number of inflammatory, autoimmune and neoplastic diseases, making them a compelling biomarker and immunotherapeutic target^2–7^. Tregs are heterogeneous in the periphery^8,9^. Despite the discovery of various Treg subtypes such as type 1 regulatory T (Tr1)^10,11^, CD8^+^ ^12,13^, CD4^+^CD25^−^LAG3^+^ ^14,15^, γδ TCR^+^^16,17^ and invariant natural killer T (iNKT)^18,19^ regulatory cells, CD4^+^FoxP3^+^ Tregs remain as the principal target of investigation in humans and mice^20,21^. CD4^+^CD25^+^FoxP3^+^ T cells in mice are suppressive^20,22^, whereas accumulating evidence suggests that CD4^+^FoxP3^+^ T cells in humans are heterogeneous in phenotype and function^23–25^. In addition to the extensive portfolio of surface markers for human Tregs, including CD25, CD127, CD45RA, ICOS and HLA-DR, sialyl lewis x (CD15s) identifies terminally differentiated effector Tregs^26^.

Although murine models for a number of pathobiological and immunotherapeutic studies are firmly established, large animal models are increasingly gaining traction. Of these, the dog recapitulates human autoimmune and neoplastic diseases remarkably well. Such diseases are spontaneous in canine patients, which have a competent immune system, and clinical presentations, treatment modalities and living environments shared with their human counterparts^27–29^. However, in-depth study of canine Tregs has been hampered by the limited availability of monoclonal antibodies (mAb) against surface antigens. Apart from the cross-reactive clones validated for canine intracellular FoxP3 (clone FJK-16s)^30,31^ and Helios (clone 226 F)^31^, anti-CD25 (clone P4A10) is the only known mAb labelling the extracellular surface of canine Tregs^32^. CD4^+^CD25^+/high^ T cells are enriched for suppressive FoxP3^+^ T cells in humans and mice^33–35^. Our previous work has shown that canine CD4^+^CD25^high^ T cells induced *in vitro* are regulatory^31^, but studies examining these cells *ex vivo* are limited in number and scope^36–38^. We therefore set out to characterise canine CD4^+^CD25^high^ T cells isolated *ex vivo*, hypothesising that they possess regulatory phenotype and function. Furthermore, we investigated the transcriptomic phenotype of Tregs in dogs and compared it with those of humans and mice on the basis of published transcriptomic data, revealing a broadly conserved Treg signature across these species and consensus transcripts encoding molecules not hitherto associated with Tregs.

## Materials and Methods

### Sample collection

This study was approved by the Clinical Research Ethical Review Board (URN 2016 1592) of the Royal Veterinary College (RVC) in the United Kingdom. Eleven healthy dogs, defined by the absence of clinical signs and a normal physical examination undertaken by a veterinarian or veterinary nurse, were recruited at the RVC. Peripheral blood samples were collected from the jugular or lateral saphenous vein in sterile fashion by a veterinarian or veterinary nurse under the Animals (Scientific Procedures) Act 1986, following informed written consent by the owners of the dogs.

### Isolation of peripheral blood mononuclear cells

Mononuclear cells were isolated from the peripheral blood by density gradient centrifugation, using Histopaque^®^-1077 (Sigma-Aldrich, Dorset, UK). Blood was diluted by an equal volume of phosphate-buffered saline (PBS; Sigma-Aldrich) with 2% v/v fetal bovine serum (FBS; Thermo Fisher Scientific, Waltham, MA, USA). The diluted blood was then layered onto an equal volume of Histopaque, before centrifugation at 400 *g* for 30 minutes at room temperature with minimal acceleration and braking. The purified peripheral blood mononuclear cells (PBMCs) were washed twice in PBS with 10% v/v FBS by centrifuging at 600 *g* for five minutes at 4 °C. After washing, cells were re-suspended in PBS with 10% v/v FBS, and counted using a haemocytometer before flow cytometric analysis. Dead cells were excluded by trypan blue staining.

### Flow cytometry

Freshly isolated PBMCs were analysed by flow cytometry using mAb against canine-specific or cross-reactive antigens (all from Thermo Fisher Scientific). Extracellular labelling was performed by incubating PBMCs for 30 minutes at 4 °C with a mixture of FITC-conjugated anti-dog CD45 (clone YKIX716.13), PerCP-eFluor^®^ 710-conjugated anti-dog CD5 (clone YKIX322.3), PE-Cy7-conjugated anti-dog CD4 (clone YKIX302.9), eFluor^®^ 450-conjugated anti-dog CD8 (clone YCATE55.9) and PE-conjugated anti-dog CD25 (clone P4A10). After washing twice with PBS, cells were incubated in eBioscience™ FoxP3/transcription factor fixation/permeabilisation buffer (Thermo Fisher Scientific) according to the manufacturer’s instructions, then labelled with APC-conjugated anti-mouse/rat FoxP3 (clone FJK-16s) for 30 minutes at 4 °C. After washing with 1x permeabilisation buffer, cells were re-suspended in 200 μL PBS before being acquired on a FACSCanto II flow cytometer (Becton-Dickinson (BD); Franklin Lakes, NJ, USA). Flow cytometric data were analysed using FlowJo^®^ software, version 7.6 (Tree Star, Ashland, OR, USA). Positive events were gated according to corresponding isotype or fluorescence minus one (FMO) controls.

### Fluorescence-activated cell sorting

Fluorescence-activated cell sorting (FACS™) was used to sort PBMCs into subpopulations for subsequent experiments. Freshly isolated PBMCs were labelled by a mixture of PerCP-eFluor^®^ 710-conjugated anti-dog CD5 (clone YKIX322.3), PE-Cy7-conjugated anti-dog CD4 (clone YKIX302.9), PE-conjugated anti-dog CD25 (clone P4A10) and Alexa Fluor^®^ 700-conjugated anti-mouse CD11b for 30 minutes at 4 °C. After washing twice with PBS, cells were stained with 4′,6-diamidino-2-phenylindole (DAPI; BioLegend, San Diego, CA, USA) at room temperature for 10 minutes prior to sorting on BD FACSAria™ II. CD4^+^CD25^high^ and CD4^+^CD25^−^ T cells were isolated from CD5^+^CD11b^−^ cells, and autologous antigen-presenting cells (APCs) were identified as CD5^−^CD11b^+^. For functional assays, CD4^+^CD25^high^ T cells were defined as the 5% of CD4^+^ T cells showing the highest CD25 expression, whereas CD4^+^CD25^−^ T cells were defined as the 20% of CD4^+^ T cells showing the lowest CD25 expression. For transcriptomic assays, CD4^+^CD25^high^ T cells were defined as the 1% of CD4^+^ T cells showing the highest CD25 expression, whereas CD4^+^CD25^−^ T cells were defined as before.

### *In vitro* suppression assay

CD4^+^CD25^high^ and CD4^+^CD25^−^ T cells sorted from the peripheral blood of healthy dogs were immediately re-suspended in complete culture medium (RPMI-1640 complemented with 10% v/v FBS, 10 mM HEPES, 100 μg/mL streptomycin, 100 U/mL penicillin and 0.5 mM β-mercaptoethanol; all reagents from Sigma-Aldrich). The responder T (Tresp) cell population (CD4^+^CD25^−^) was stained with CellTrace™ violet proliferation dye according to the manufacturer’s instructions (Thermo Fisher Scientific), and seeded into a 96-well plate at a density of 1–5 × 10^4^ cells per well. The suppressor cell population (CD4^+^CD25^high^) was co-cultured with Tresp cells at a ratio (Treg:Tresp) of 1:1 and/or 1:2. A population of autologous CD5^−^CD11b^+^ monocytes at a proportion of 1/5 of that of Tresp cells were also seeded into each well, as APCs. The mixed cell culture contained a total volume of 200 μL with 2.5 μg/mL concanavalin A (ConA) (Sigma-Aldrich) and was incubated for 96 hours at 37 °C, with 5% CO_2._ Three control groups were set up in the same fashion, including un-stimulated Tresp alone, stimulated Tresp alone and CD4^+^CD25^−^ co-cultured with Tresp.

### RNA extraction

CD4^+^CD25^high^ and CD4^+^CD25^−^ T cells sorted from the peripheral blood of five healthy dogs were immediately re-suspended in RNA Bee (AMS Biotechnology, Abingdon, UK) at a density of 2 × 10^6^ cells/mL. Two hundred microlitres of chloroform (Sigma-Aldrich) per millilitre of RNA Bee suspension were added, before thorough admixture, transfer to a 2 mL MaXtract High Density tube (QIAGEN, Hilden, Germany), and incubation on ice for three minutes. The tube was then centrifuged at 12,000 *g* for 15 minutes at 4 °C. After centrifugation, the upper aqueous layer was carefully transferred to a 1.5 mL DNase/RNase-free Eppendorf Tube^®^ (Eppendorf, Stevenage, UK), before being mixed completely with an equal volume of 100% ethanol (Sigma-Aldrich). The mixture was then transferred into a Zymo-Spin™ IC column on top of a collection tube and centrifuged according to the manufacturer’s instructions (Direct-zol™ RNA MicroPrep Kit, Zymo Research, Irvine, CA, USA). All samples were treated with DNase I during extraction; the final product was eluted in 6–10 μL of DNase/RNase-free water.

### Library construction and sequencing

SMARTer^®^ Universal Low Input RNA Kit (Clontech, California, USA) was used to construct the complementary (c) DNA library at the Oxford Genomics Centre, University of Oxford (Oxford, UK). RNA was converted to cDNA using Oligo (dT) primers and adapters, followed by PCR amplification. The cDNA library was then sheared into short fragments using a Covaris S220 Focused-Ultrasonicator (Thermo Fisher Scientific) for subsequent random shotgun Illumina sequencing. The 75-bp, paired-end sequencing was performed on the prepared DNA libraries, using the HiSeq. 4000 System (Illumina, San Diego, CA, USA) at the Oxford Genomics Centre. Samples were loaded onto the clustered sequencing Flow Cell, which was then primed with sequencing by synthesis (SBS) reagents and hybridised by Read 1 and Read 2 primers. The run was recorded by HCS 3.4.0 (Illumina).

### Read processing and expression quantification

Sequencing reads were trimmed using Skewer (version 0.1.125) to remove the adapter and anchor sequences added during library construction and sequencing. Trimmed transcript reads were mapped to the canine genome, CanFam3.1 (Ensembl Genes, release 91), using HISAT2 (version 2.0.0-beta). The uniquely mapped read pairs were quantified using featureCounts (version 1.5.0), and annotated using the same canine genomic data. Mapping metrics were generated using Picard Tools (version 1.92). The metrics and variants for assessing read distribution, biotype distribution and mapped transcripts were generated using R packages (version 3.4.2) with in-house scripts. Read counts were all converted to transcripts per million (TPM) to normalise sequencing depth and gene lengths.

### Differential expression analysis

Transcripts differentially expressed between canine CD4^+^CD25^high^ and CD4^+^CD25^−^ T cells were identified using Bioconductor package edgeR (Bioconductor version 3.6), with fold change (FC) values and statistical significance, the latter of which was represented by false discovery rate (FDR). R version 3.4.2 was used to conduct principal component analysis (PCA) and volcano plots.

### Ingenuity pathway analysis

Differentially expressed transcripts (FDR < 0.05) with FC and FDR values were input into the software Ingenuity Pathway Analysis (IPA; Ingenuity Systems Inc., Redwood City, CA, USA) to identify biological pathways affected by the altered expression of these transcripts (|Z| score ≥ 2).

### Reverse transcription and quantitative PCR

Purified total RNA was converted to cDNA by performing reverse transcription (RT), using the Precision nanoScript™ 2 Reverse Transcription Kit (Primerdesign, Southampton, UK). One reaction of 20 μL volume in total contained RNA template (up to 2 μg), combined Oligo (dT) and random nonamer primers, nanoScript™ 2 Buffer, dNTP mix, nanoScript™ 2 enzyme and RNase/DNase free water. The reaction included an annealing step of 65 °C for five minutes, then immediate cooling on ice, followed by an extension step at room temperature for five minutes and 42 °C for 20 minutes, then 75 °C for 10 minutes. The abundance of transcripts of interest was then measured by quantitative (q) PCR, using cDNA as reaction template, according to the manufacturer’s instructions. Primers specific to each transcript were all from the Taqman^®^ Gene Expression Assays (GEAs) (Thermo Fisher Scientific), targeting *fam129a* (Cf02724989_m1), *lmna* (Cf02678125_g1), *cadm1* (Cf02645230_m1), *anxa2* (Cf02734571_gH), *ctsz* (Cf02661948_m1), *actn4* (Cf02689744_g1), *csf1* (Cf01094425_m1), *hip1* (Cf02698307_m1), *galm* (Cf02648153_m1), *pou2f2* (Cf00922171_g1), *frmd4b* (Cf02646908_m1), *il2ra* (Cf02623133_m1), *foxp3* (Cf02741700_m1) and *ikzf2* (Cf00915981_m1). Two reference transcripts, *ubc* encoding CG11624-PA, isoform A and *sdha* encoding succinate dehydrogenase flavoprotein subunit, were selected following validation by means of the Primerdesign Dog geNorm™ Kit. The relative expression of the target transcript was calculated using Pfaffl’s model^39^ as below:

$$Relative\,expression=\,\frac{{({E}_{TAR})}^{\Delta {C}_{qTAR}(Control-Sample)}}{{({E}_{REF})}^{\Delta {C}_{qREF}(Control-Sample)}}$$

E represents E value; TAR, target transcript; REF, reference transcript; Control, CD4^+^CD25^−^ cells; Sample, CD4^+^CD25^high^ cells. The relative expression ratio calculated by this equation indicated the FC of the target transcript abundance in the sample population when compared to that of the control population.

### Interspecies comparisons

To compare the transcriptomic profiles of canine CD4^+^CD25^high^ T cells across species with those of human and murine Tregs, published resources were used. The selected human and murine studies^22,40^ used different analytical methods from those in this study, but were the most comprehensive in the literature and conducted on freshly isolated Tregs in comparison to CD4^+^CD25^−^ T cells. Raw transcriptomic data of the published human and murine studies were analysed following the same pipeline as for canine CD4^+^CD25^high^ T cells, with respective genomic information. The data were processed using the web-based bioinformatics platform *Galaxy*^41^. Similarity scores were calculated using R OrderedList^42^ (version 1.48.0), to determine the number of shared transcripts between two species in the first n consensus transcripts, which were ordered by differential expression FC values. A similarity score was yielded, in which transcripts received higher weight the closer they were to the top or bottom end of the ordered list. Similarity scores for n = 100, 150, 200, 300, 400, 500 and 750 transcripts were reported, respectively. Statistical significance was assessed for each of the similarity scores, by comparing with a null distribution generated by randomly scrambling the order of the transcripts.

### Statistical analysis

Summary data are shown as mean ± standard error of the mean (SEM). Statistical analysis was performed using GraphPad Prism version 7 (GraphPad Software, La Jolla, CA, USA).

## Results

### Freshly isolated canine CD4^+^CD25^high^ T cells are enriched for FoxP3

**Figure 1:**
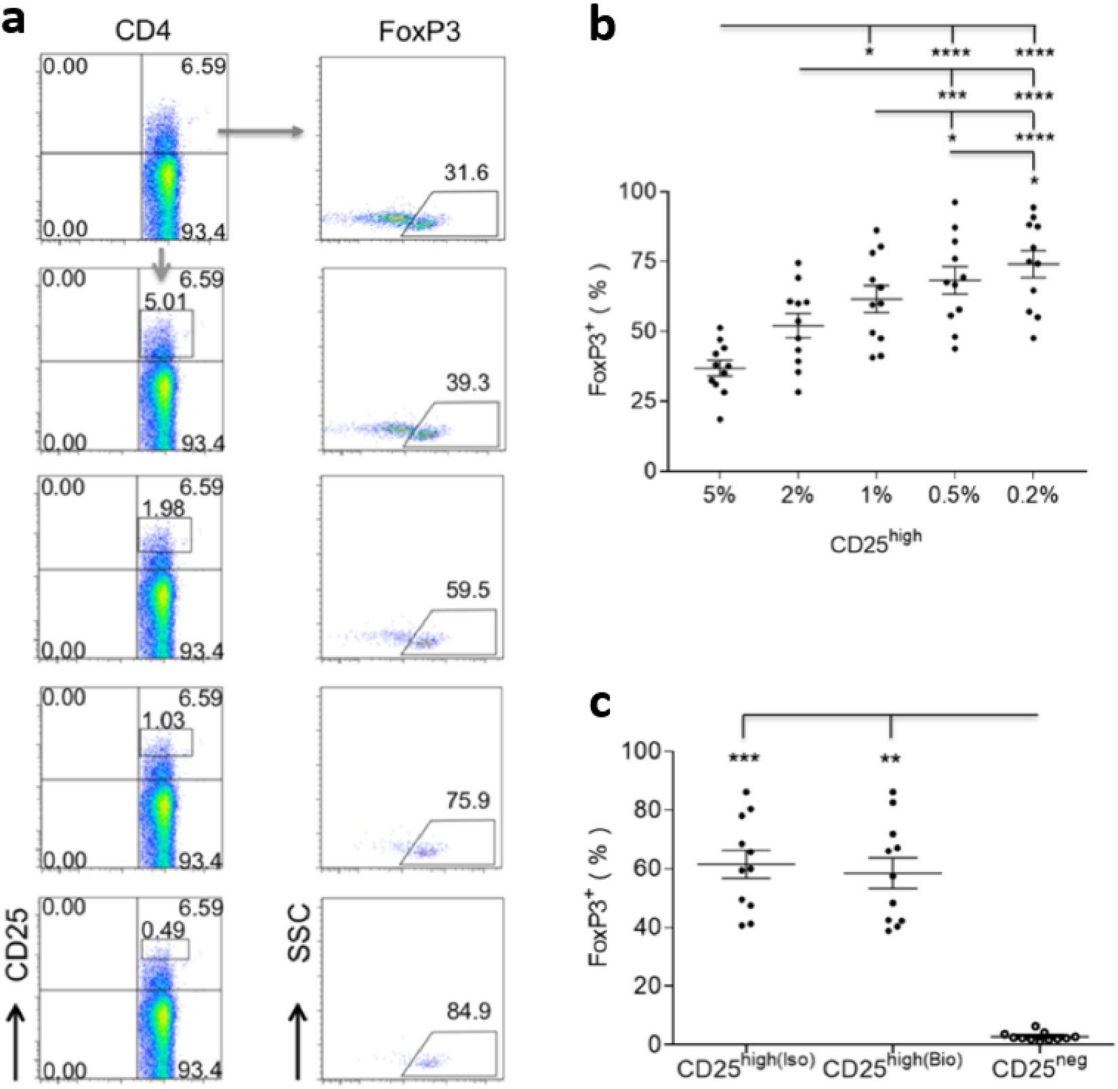
CD4^+^CD25^high^ T cells isolated *ex vivo* are enriched for FoxP3. (**a**) Representative flow cytometric plots showing that proportional expression of FoxP3 increased with increasing CD25 expression by CD4^+^ T cells from the highest 5% to the highest 0.5% of one healthy dog (all CD4^+^CD25^+^ T cells in this figure were analysed as CD45^+^CD5^+^CD8^-^CD4^+^CD25^+^, following a cascaded gating strategy). (**b**) Scatter dot plot summarising the increasing proportional expression of FoxP3 (mean ± SEM) among CD4^+^ T cells of 11 healthy dogs, with increasing CD25 expression from the highest 5% to the highest 0.2%. (**c**) Summary scatter dot plot comparing the higher proportional expression of FoxP3 in top 1% of CD25^high^ cells, in which gating was determined by the corresponding isotype control (iso) or biological negative control (bio; CD25^−^). No significant difference was found in CD25^high^ cells between the two gating methods. Statistical significance in (**b**,**c**) was analysed by one-way ANOVA, followed by Dunn’s multiple comparisons test (****p < 0.0001, ***p < 0.001, **p < 0.01, *p < 0.05).

To test the hypothesis that freshly isolated canine CD4^+^CD25^+^ T cells have a regulatory phenotype, PBMCs of 11 healthy dogs were labelled with a mAb panel incorporating all markers of canine Tregs to date. When the CD25 gate was moved upwards to incorporate increasing CD25 expression per cell, from the highest 5% to the highest 0.2%, the proportion of FoxP3^+^ cells significantly increased from 36.89 ± 2.79% to 74.07 ± 4.81%, suggesting that *ex vivo* CD4^+^CD25^high^ T cells were enriched for FoxP3 (Fig. 1a,b).

The top 1% of CD4^+^CD25^+^ T cells were selected for subsequent phenotypic characterisation, balancing the enrichment for FoxP3 (61.59 ± 4.76%) with the need to isolate sufficient numbers. The proportional expression of FoxP3 in CD4^+^CD25^high^ T cells was compared to CD4^+^CD25^−^ cells of the same dogs, the latter selected by gating the 20% of CD4^+^ T cells showing the lowest CD25 expression. FoxP3^+^ cells in the CD25^high^ fraction were gated in two ways, making a comparison with either the corresponding isotype control or the paired CD25^−^ population (a negative biological control). The two gating methods yielded similar results: CD25^high^ T cells had significantly greater FoxP3 expression than CD25^−^ T cells from the same dogs (Fig. 1c).

### Freshly isolated canine CD4^+^CD25^high^ T cells are suppressive *in vitro*

**Figure 2:**
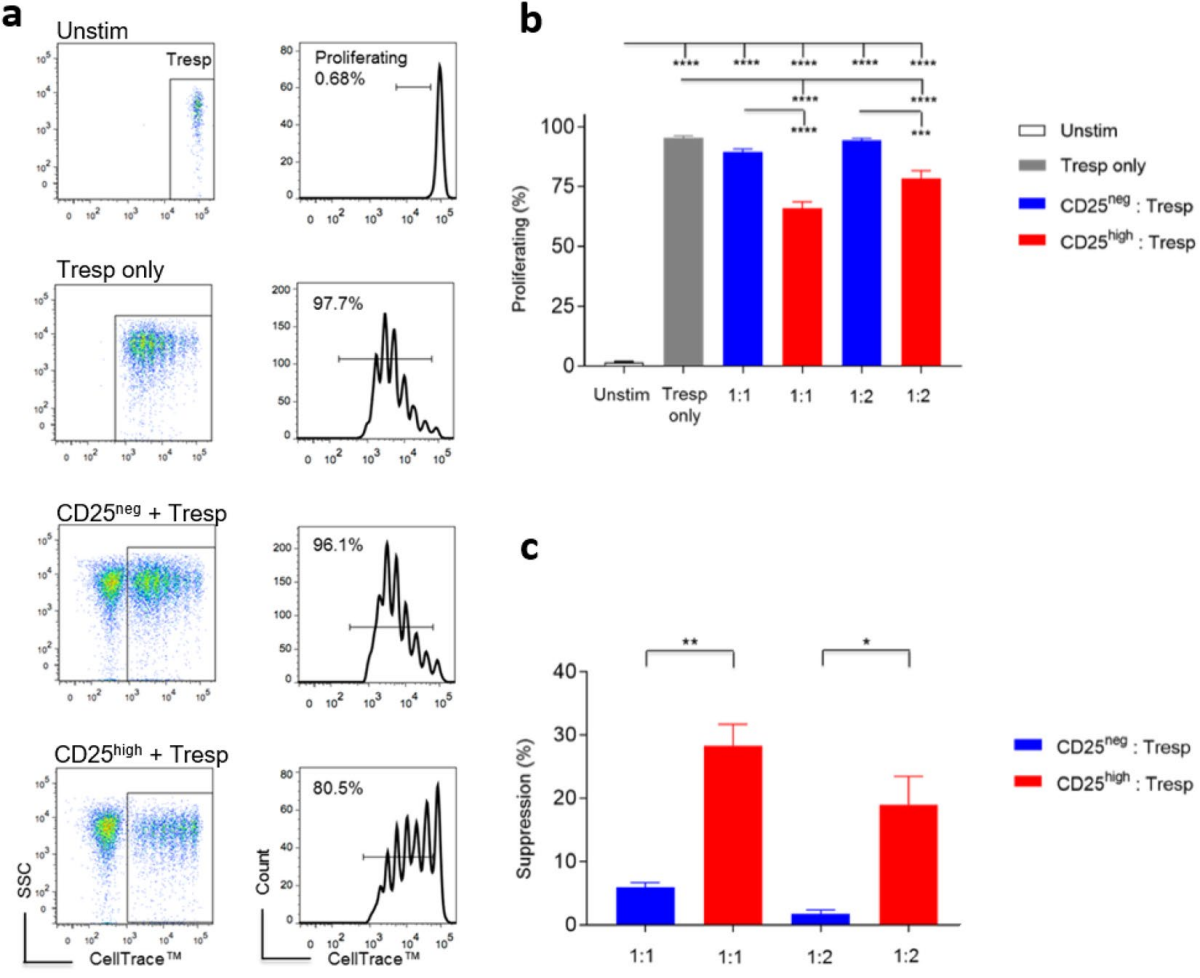
CD4^+^CD25^high^ T cells isolated *ex vivo* are suppressive *in vitro*. (**a**) Representative flow cytometric plots showing the proliferation of pre-labelled Tresp cells analysed in flow cytometry after a 96-hour incubation. Suppressor and responder cells were co-cultured at the ratio of 1:1. Tresp T cells of the four groups were gated following the same cascaded strategy: from live cells, to lymphocytes, to Tresp cells, followed by measurement *via* proportional proliferation. (**b**) Summary bar charts showing the proliferation of Tresp cells post 96-hour incubation (mean ± SEM), measured by means of proportional proliferation, at both 1:1 and 1:2 suppressor: responder ratios (five independent experiments). Statistical significance was analysed by one-way ANOVA, followed by Holm-Sidak’s multiple comparisons test. (**c**) Summary bar charts showing the percent suppression mediated by the suppressor population, normalised to parallel stimulated Tresp cells ((proliferating % of Tresp only − proliferating % of co-cultured Tresp)/(proliferating % of Tresp only) × 100 (five independent experiments; mean ± SEM). Statistical significance was determined by means of a paired *t* test (****p < 0.0001, ***p < 0.001, **p < 0.01, *p < 0.05).

Freshly isolated CD4^+^CD25^high^ T cells suppressed conventional CD4^+^CD25^−^ T cell proliferation, as indicated by reduced cell divisions at a ratio of 1:1 or 1:2 in the presence of autologous monocytes (CD5^-^CD11b^+^) and ConA (Fig. 2). Our findings therefore confirmed the suppressive function of *ex vivo* canine CD4^+^CD25^high^ T cells. Given their regulatory phenotype and function, we then hypothesised that canine CD4^+^CD25^high^ T cells have a transcriptomic profile characteristic of Tregs.

### Canine CD4^+^CD25^high^ T cells possess the transcriptomic signature of Tregs

**Figure 3:**
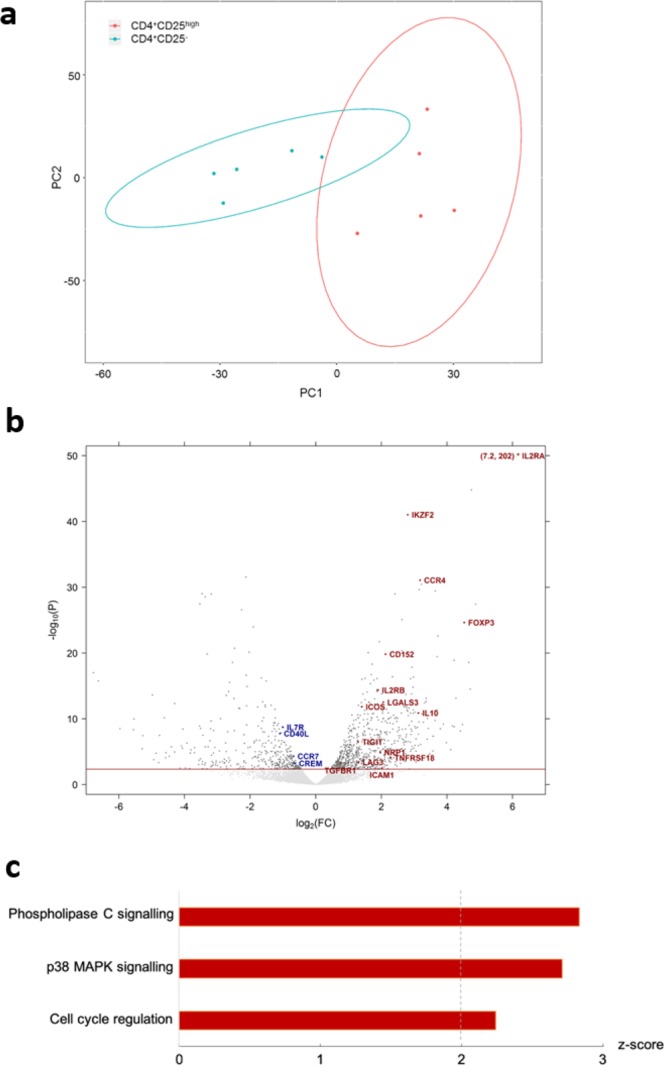
CD4^+^CD25^high^ T cells possess the transcriptomic signature of Tregs. (**a**) Genome-wide expression data of 9,476 transcripts of five CD4^+^CD25^high^ and paired CD4^+^CD25^−^ T cell samples isolated *ex vivo* from five healthy dogs were plotted by PCA, with the principle component 1 (PC1) of 29.8% and PC2 of 17.9%. (**b**) Expression data of differentially expressed transcripts of the same five CD4^+^CD25^high^ *versus* paired CD4^+^CD25^−^ T cell samples as in (**a**), revealed by volcano plot. Threshold line in red indicates FDR = 0.05, and each dot represents one transcript. Transcripts above threshold were differentially expressed, with Tregs-specific transcripts annotated with symbols. For better visualisation, transcript symbols were designated in upper case. The transcript *il2ra* was also designated with coordinates, owing to its striking values for FC and statistical significance, both off scales. (**c**) Stacked bar charts showing z-scores of enriched biological pathways identified by IPA, with red colour representing activated status. The dashed line highlights a z-score of 2; absolute values ≥ 2 indicate high consistency of expression direction between the input transcripts and IPA knowledge database. All highlighted pathways were statistically significant (*p* < 0.05).

We conducted RNA-seq on freshly isolated CD4^+^CD25^high^ and CD4^+^CD25^−^ T cells. PCA analysis revealed distinct expression signatures of the two cell types (Fig. 3a). A volcano plot confirmed the distinction and suggested a Treg-like phenotype of CD25^high^ T cells, which preferentially expressed nearly all of the known Treg-specific transcripts, such as *il2ra*, *foxp3*, *ikzf2*, *ctla4*, *il10*, *lgals3*, *tigit*, *nrp1*, *lag3*, *icam1* and *tnfrsf18*^43,44^ (Fig. 3b).

### Ingenuity pathway analysis of canine CD4^+^CD25^high^ T cells

Pathway analysis further consolidated functional annotations of the CD25^high^ T cell expression signature in comparison to CD25^−^ T cells, which identified three pathways associated with development and function of Tregs to be activated, namely phospholipase C signalling, p38-mitogen activated protein kinase (MAPK) signalling and cell cycle regulation (Fig. 3c).

### A Treg-specific expression signature is conserved in humans, mice and dogs

**Figure 4:**
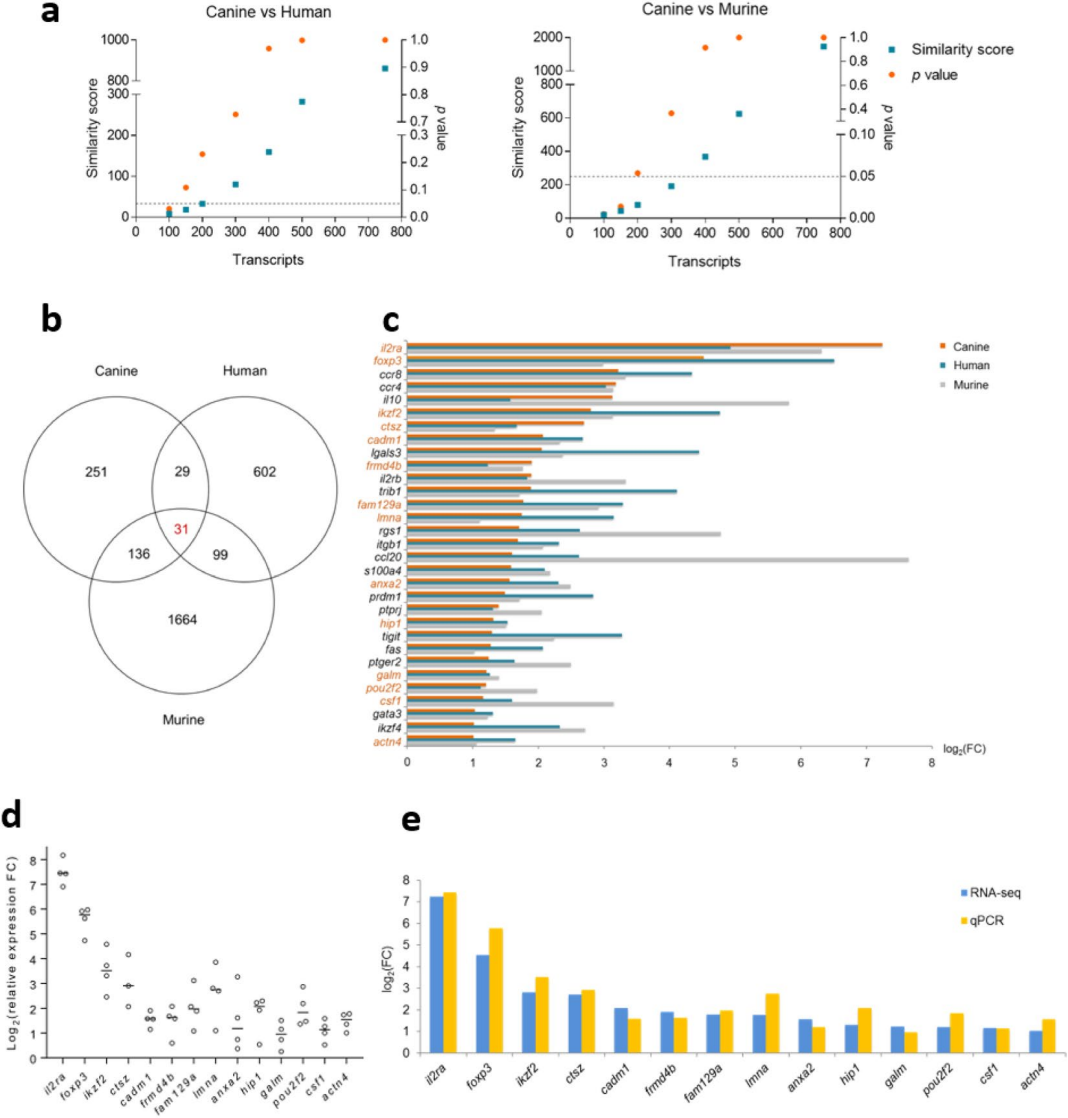
A Treg-specific transcriptomic signature is conserved in humans, mice and dogs. (**a**) Similarity score analysis measured the resemblance between differentially expressed transcripts of canine CD4^+^CD25^high^ T cells with those of human and murine Tregs, on the basis of 772 consensus transcripts. Similarity score was calculated using the ranked top 100, 150, 200, 300, 400, 500 and 750 transcripts, respectively, with an accompanying *p* value. The dashed line indicates *p* = 0.05. (**b**) Venn diagram showing highly enriched transcripts (with more than two-fold preferential expression) consensus between canine CD4^+^CD25^high^ T cells and, human and murine Tregs. (**c**) Stacked bar charts showing the 31 consensus transcripts conserved in all three species, with corresponding FC values in log_2_ format. Transcripts selected for RT-qPCR validation are highlighted in orange. (**d**) Scatter plots showing relative expression FC values of transcripts validated by RT-qPCR, plotted in log_2_ format. The line indicates median value of the three or four sample replicates. (**e**) Stacked bar charts showing expression FC values of transcripts preferentially expressed by canine CD4^+^CD25^high^ T cells compatible between RNA-seq and RT-qPCR detection, plotted in log_2_ format.

We compared Treg-specific transcriptomic signatures between species using similarity scores, which revealed a resemblance of canine CD4^+^CD25^high^ T cells to both human and murine Tregs for the top 100 most differentially expressed transcripts (Fig. 4a). Of interest, human and murine Tregs showed no significant similarity (Supplementary Fig. S1). Thirty-one transcripts highly enriched in Tregs (FC > 2) were consensus in all three species (Fig. 4b). Among them, six transcripts encode the Treg signature molecules *il2ra*, *foxp3*, *il10*, *ikzf2*, *lgals3*, and *tigit*^43,44^. Thirteen transcripts, namely *ccr8*, *ccr4*, *il2rb*, *trib1*, *rgs1*, *itgb1*, *ccl20*, *s100a4*, *prdm1*, *fas*, *ptger2*, *gata3* and *ikzf4*, are associated with development and function of Tregs^44–50^. The remaining 12 transcripts have not been associated with Tregs previously (Fig. 4c). Preferential expression of 11 transcripts not hitherto related to Tregs was confirmed by RT-qPCR, together with *il2ra*, *foxp3* and *ikzf2* as positive controls; primers for canine *ptprj* were unavailable at the time of this study, precluding confirmation of this transcript (Fig. 4d). All of the 14 transcripts examined by RT-qPCR showed greater expression in canine CD4^+^CD25^high^ T cells compared to CD4^+^CD25^−^ T cells, with FC values comparable to those detected by RNA-seq (Fig. 4e).

## Discussion

We have shown that canine CD4^+^CD25^high^ T cells isolated *ex vivo* have the transcriptomic signature of Tregs, reconciling with their regulatory phenotype and function. Moreover, the transcriptomic signature of canine CD4^+^CD25^high^ T cells resembled those of human and murine Tregs, consistent with our view that they represent Tregs.

Apart from FoxP3 and other Treg signature molecules, we found that the canine CD4^+^CD25^high^ T cells expressed transcripts encoding transcription factors specific to pro-inflammatory T helper (Th) cells in greater abundance than CD4^+^CD25^−^ T cells. For instance, CD25^high^ T cells preferentially expressed *gata3* and *irf4* of Th2 cells^51–53^ and, *batf*, *ikzf3*, *ikzf4* and *rorα* of Th17 cells^54–57^. A trivial explanation of this phenomenon was enrichment of effector Th cells within the CD25^high^ T cells, which were not exclusively FoxP3^+^ and likely to be contaminated by Th cells. Healthy dogs are exposed to environmental antigens at mucosal surfaces on a continuous basis, with subsequent polarisation of a proportion of the local T cells and escape of these cells into the peripheral blood. An alternative explanation was that some of the peripheral Tregs themselves expressed Th-specific transcription factors, as has been previously documented^25,58–62^. The CD4^+^CD25^high^ T cells also expressed a number of homing receptor transcripts at greater abundance than the CD4^+^CD25^−^ T cells. For instance, CD25^high^ T cells preferentially expressed Th2-associated chemokine receptor transcripts *ccr3*, *ccr4* and *ccr8*^63–65^, in line with the greater expression of Th2 transcription factor transcripts *gata3* and *irf4*. Other chemokine receptors enriched in canine CD25^high^ T cells are expressed by human and murine Tregs resident in various tissues and organs, i.e. CXCR6 and CCR3 in adipose tissue^66^, CCR2, CCR5 and CXCR3 in pancreas^67^, CCR4 in skin^68,69^ and, CCR2, CCR5 and CCR8 in muscle^70^. In contrast, CD25^high^ T cells expressed three transcripts encoding naïve T cell homing molecules CD62L (L-selectin), CCR7 and IL7R^71–74^ in lower abundance. Trafficking of Tregs to peripheral lymphoid and non-lymphoid niches is critical to their functions in homeostasis, autoimmune disease and cancer in humans and mice, and expression of homing receptors may vary with developmental stage and target locations of Tregs^68,70,75–80^. Single-cell RNA-seq would be required to distinguish whether these differential expression patterns were attributable to contaminant Th cells or to *bona fide* Tregs. Nevertheless, these data raise the intriguing possibility of ectopic expression of Th-specific transcripts by Tregs in dogs, as in other species: for instance, human Tregs isolated *ex vivo* from healthy donors express *gata3* and *ccr4* of Th2 cells^25^, and murine Tregs incorporate *irf4* to suppress Th2 response^58^.

Pathways associated with the development and function of canine Tregs were identified in our dataset. A cascade of signal transduction pathways is engaged upstream and downstream of FoxP3, dedicating Tregs to lineage-specific commitment^81–88^. Phospholipase C signalling is a critical transduction pathway downstream of TCR activation in Tregs, and its defect causes profound autoimmune lesions in mice^89^. The dominant mediator phospholipase C produces secondary messenger molecules 1,4,5-trisphosphate (IP_3_) and diacylglycerol (DAG)^90–92^. IP_3_ activates calcium flux, which then triggers the transcription factor nuclear factor of activated T cells (NFAT) to interact with FoxP3^89,91^. DAG functions in a cascade upstream of p38-MAPK signalling, which regulates the cell cycle and is indispensable in the induction of anergy and maintenance of Treg suppressive function^93^. The upregulation of phospholipase C, p38-MAPK and cell cycle regulation pathways in canine Tregs accords with these observations.

We interrogated expression signatures of Tregs across species, reasoning that similarity of transcripts would speak to their core function in Tregs. Canine Tregs resembled both human and murine Tregs, yielding 31 common differentially expressed transcripts. More than half of the 31 consensus transcripts encode Treg-specific molecules, indicative of interspecies conservation of Treg signature. Of the 12 transcripts not hitherto related to Tregs, *hip1* has potential immunoregulatory relevance. Hip1 is a serine hydrolase protein embedded in cell envelopes of *Mycobacterium tuberculosis*, which reside intracellularly in macrophages and dendritic cells (DCs) of the host, evading immune responses by impeding functions of these primary APCs using Hip1^94–97^. First, *M*. *tuberculosis* deactivates Toll-like receptor 2 and MyD88-dependent pathways *via* Hip1, reducing activation and cytokine production of macrophages and DCs^94,96^. Second, *M*. *tuberculosis* disrupts interactions between CD4^+^ T cells and APCs through GroEL2, a product of Hip1 hydrolysis^95,97^. Therefore, Hip1 may be another mechanism by which Tregs negatively modulate APCs. Fam129a and Alpha actinin-4 encoded by *fam129a* and *actn4* inhibit cell apoptosis^98,99^, and Cathepsin Z, encoded by *ctsz*, promotes angiogenesis and metastasis^100,101^. These three proteins could potentially be blocked by specific mAb to attenuate the number and function of Tregs in the cancer microenvironment. The remaining eight transcripts are involved in T cell activation: protein products of *cadm1*, *frmd4b*, *lmna*, *anxa2*, *galm*, *pou2f2*, *csf1* and *ptprj* may directly or indirectly enhance TCR signalling or interaction of T cells with APCs^102–109^. Single cell RNA-seq would be required to further explore the significance of these transcripts to Tregs, along with confirmation of differential expression of their protein products and their role in suppressive function, if any.

In conclusion, we have characterised the phenotype, function, and transcriptomic signature of canine Tregs. We have delineated a core set of 31 transcripts that show differential expression by the Tregs of three mammalian species, including humans. More than half of these transcripts have been previously associated with Tregs in mice and humans. However, 12 transcripts have hitherto not been associated with Tregs in any species, prompting further questions about their role in this cellular context. This comparative approach is a powerful tool in generating hypotheses that may yield fresh mechanistic insights or novel immunotherapeutic targets in this important, yet elusive, area of immunology.

## Supplementary information

Phenotypic characterisation of regulatory T cells in dogs reveals signature transcripts conserved in humans and mice

## Data Availability

Raw and processed canine RNA-seq data of this study have been deposited to Gene Expression Omnibus (GEO), accession number GSE132068.
