# Supplementary material for: Phenotypic characterisation of regulatory T cells in dogs reveals signature transcripts conserved in humans and mice
Source: Sci Rep. 2019 Sep 17;9:13478. doi: 10.1038/s41598-019-50065-8 (PMC6748983; doi:10.1038/s41598-019-50065-8)
Supplement: Supplementary file 1 — Phenotypic characterisation of regulatory T cells in dogs reveals signature transcripts conserved in humans and mice [file 41598_2019_50065_MOESM1_ESM.pdf]

# Phenotypic characterisation of regulatory T cells in dogs reveals signature transcripts conserved in humans and mice

**Ying Wu[1, ¶], Yu-Mei Chang[1], Anneliese J. Stell[1], Simon L. Priestnall[1], Eshita Sharma[2], Michelle R. Goulart[1, ¶], John Gribben[3], Dong Xia[1, §], Oliver A. Garden[1, 4, §, *]**

[1]Royal Veterinary College, London, UK; [2]Wellcome Centre for Human Genetics, University of Oxford, Oxford, UK; [3]Barts Cancer Institute, Queen Mary University of London, London, UK; [4]School of Veterinary Medicine, University of Pennsylvania, Philadelphia, PA, USA

[¶]Current address: Y.W.: School of Veterinary Medicine, University of Pennsylvania, Philadelphia, PA, USA; M. R. G.: Barts Cancer Institute, Queen Mary University of London, London, UK

[§]Co-senior authorship

[*]Corresponding author: O.A.G. (email: ogarden@upenn.edu)

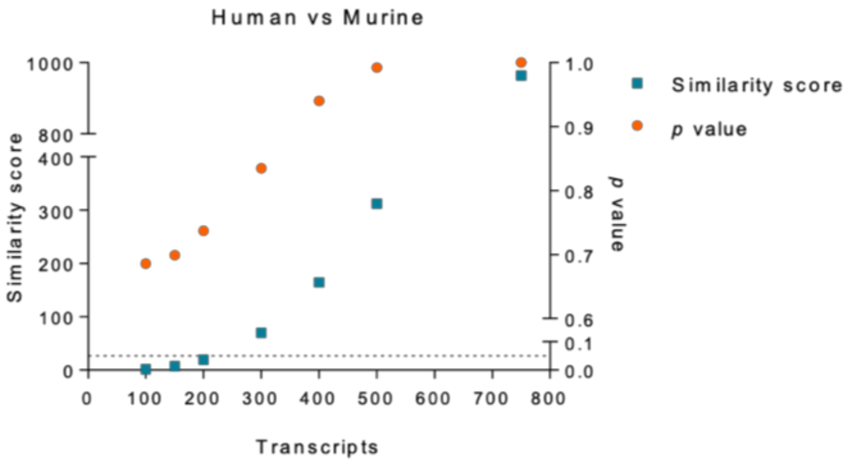


**Supplementary Figure S1: Similarity score of human *versus* murine Treg transcriptomic data**

Similarity score analysis was performed on the basis of same 772 consensus transcripts as in Figure 4a. Similarity score was calculated using the ranked top 100, 150, 200, 300, 400, 500 and 750 transcripts, respectively, with an accompanying *p* value. The dashed line indicates $p = 0.05$.
